# Editorial: Immunovirology in aquatic animals

**DOI:** 10.3389/fimmu.2025.1655117

**Published:** 2025-08-26

**Authors:** Carolina Johnstone, Elena Chaves-Pozo

**Affiliations:** ^1^ Physiology and Welfare of Marine Species Group (PHYSIS), Centro Oceanográfico de Málaga, Instituto Español de Oceanografía (COMA-IEO), Consejo Superior de Investigaciones Científicas (CSIC), Málaga, Spain; ^2^ Physiology and Welfare of Marine Species Group (PHYSIS), Centro Oceanográfico de Murcia, Instituto Español de Oceanografía (COMU-IEO), Consejo Superior de Investigaciones Científicas (CSIC), Murcia, Spain

**Keywords:** viral infectious disease, marine virus, viral zoonosis, antiviral, aquaculture, viral immunity

Aquatic ecosystems are home to invertebrate and vertebrate animal species and they provide important natural resources, including food. Protein-rich aquatic animal foods contribute to global food security and nutrition. Over the last decades the production of fish food through aquaculture has surpassed that of fisheries (1). One of the challenges currently faced by aquaculture is viral infectious diseases, which can affect product quality and cause harvest losses. This Research Topic collects in its first volume part of the current knowledge on viral immunology in freshwater and marine fish species, from high commercial value species to species of interest for aquaculture diversification. 

Global warming in aquatic ecosystems alters the distribution of species contributing to the spread and emergence of new viruses. The stress response triggered in all living specimens by environmental changes, including pollution, pushes viral emergence to its peak. In this sense, mitigation tools will be needed in the near future and are worth investigating as in the case of iron nanoparticles, which have been found to modulate gene expression in fish (Kumar et al.). In addition, a soluble recombinant glycoprotein nanoparticle of IHNV was constructed and proved to be stable and immunogenic *in vitro* (Ahmadivand et al.), opening the possibility of using self-assembling ferritin nanocages as vaccine platforms for fish viral antigens. This volume also presents foundational research on viral diseases that affect global salmonid aquaculture. Extensive cell surface modulation in infectious salmon anemia virus (ISAV)-infected erythrocytes of Atlantic salmon was found to be directly linked to the viral surface glycoprotein hemagglutinin esterase, which binds to sialic acid, acting as a viral receptor-destroying enzyme (Fosse et al.). Infectious hematopoietic necrosis virus (IHNV) is another lethal viral pathogen that affects the global trout and salmon aquaculture industry, for which no commercial vaccine is available. A transcriptomic approach in salmonid red blood cells was performed to assess differentially expressed genes that may affect the viral propagation during the early phases of infection with piscine orthoreovirus 1 (PRV-1), which causes heart and skeletal muscle inflammation (HSMI) (Tsoulia et al.). Transcriptomics were also employed to assess the physiological status of a species of interest for Mediterranean aquaculture diversification: the Shi drum. This study established that high culture densities could hinder the control of nervous necrosis virus (NNV) outbreaks (Garcia-Beltran et al.). Since having an overview of the immune response is interesting for compiling knowledge on specific fish viral pathogens, this volume also presents a review of the recent advances in the immune response to Tilapia lake virus (TiLV) (Kembou-Ringert et al.), a novel lethal RNA virus that represents a threat to tilapia aquaculture, one of the top ten products of fisheries and aquaculture (1). Also, viral infectious diseases can affect global aquaculture production when they compromise meat quality or cause secondary infections. This is the case of the self-limiting chronic Lymphocystis disease, which is characterized by the growth of papilloma-like nodules on the skin and fins of infected fish. The differentially expressed genes in the infected skin nodules associated with the lymphocystis disease virus (LCDV) were investigated through transcriptomics in flounder (*Paralichthys olivaceus*), a species of economic importance in Asian countries (Zhang et al.). In gilthead seabream (*Sparus aurata*), a species of economic importance for Mediterranean aquaculture, the immune genes involved in the protection induced by a DNA vaccine based on the major capsid protein of an LCDV were investigated through an array platform (Leiva-Rebollo et al.). Acipenserid herpesvirus 2 (AciHV-2), another DNA virus, causes a fatal disease in juvenile white sturgeons (*Acipenser transmontanus*) and was found to affect the outcome of secondary bacterial infections (Quijano Cardé et al.). Finally, poly(I:C) (polyriboinosinic polyribocytidylic acid), an analogue of double stranded viral RNA that is used as an adjuvant and is known to induce viral immune responses in fish, was employed to assess the antiviral response in species such as lumpfish, a species that is important for the control of sea lice in salmon aquaculture, or the gadiform species *Lota lota*, whose population is decreasing.

The prevention and treatment of aquatic animal viral diseases requires basic research in comparative immunology to understand the host response in fish, a group of animals with evolutionary diversification. Our knowledge of viral immunology is essential for developing treatments and vaccines against infectious viral diseases in fish, which cause high mortality rates and significant economic losses for aquaculture producers. This first volume provides a foundation of knowledge on the cellular and molecular mechanisms underlying fish innate and adaptive immunity to different infectious viral diseases and explores novel treatments.
